# Cancer Research in the Registrar's Room

**Published:** 1909-09-11

**Authors:** 


### The Hospital

A Journal of

The Medical Sciences and Hospital Administration.

New Series. No. 133, Vol. V. [No. 1205, Vol. XLVI.] SATURDAY, SEPTEMBER 11, 1909.

### Cancer Research in the Registrar's Room.

Cancer research promises to become one of the fashionable studies of the rising generation of medical men. It is one of the most promising signs of progress that fashion should have fixed on so fine and useful an object for its favourite, although it may safely be premised that the good that will result from such research will not be due to the fact that fashion has had anything whatever to do with it. Nevertheless it is gratifying to note that yearly a larger percentage of recently qualified men devote themselves to the elucidation of this problem. Twenty years ago a few enthusiasts occupied the field where there are now dozens of patient observers and keen pathologists actively at work. This is as it should be, for the more men devote their energies to the work the greater is the likelihood of something tangible resulting from it. The wider the field is made, and the more diverse are the methods of research, the greater is the probability of ultimate success.

At present the pathological side appears to have put the clinical wholly into the shade. We regret that this should be so, for we are convinced that a great deal may be done by patient clinical investigation. By clinical investigation we understand the widest and fullest interpretation that can be given to the term "bedside work," but it must be remembered that there are branches of clinical work which cannot be readily performed in the ward alongside the patient's bed. One of these branches—one hitherto neglected so far as the cancer problem is concerned—is the statistical side. It is high time that some research student, possessed of a training in dealing with statistics, should sacrifice his pathologico-bacteriological yearnings, and devote a couple of years to a close study of the statistics of cancer. On a small scale much has already been done. One of the finest contributions to the literature of carcinoma mammæ, for example, is Finsterer's exceptionally full and able record of all Bilroth's cases and their after history; a masterly piece of work which is by no means so well known to English surgeons as it deserves to be. Another instance, on a still smaller scale, is furnished by Mancrede's article in the current *Annals of Surgery* on "The After Results of Excision of the Scapula in cases of Malignant Disease." From the surgeon's point of view such collected observations are of the utmost importance. From the point of view of the research student they offer a wide field for original investigation. The registrars' bookshelves in all our larger hospitals hold a mass of valuable and interesting statistical material, which should be made more generally available than it is at present. Here are preserved many very valuable records which demand collation, and which should prove most useful to the expert statistician. What is wanted is work somewhat on the lines adopted by Finsterer and Nancrede. Each case should be tabulated, and, where patients are alive, they should be invited to submit themselves to examination by an independent investigator. Individual statistics and reports prepared by operators themselves, who take into account only their own series, and who are inevitably biassed, no matter how wide their outlook or how unprejudiced their observations, in favour of their own cases, can hardly be reckoned equal in value to such independently investigated analyses. The work such as we suggest is by no means easy, and the sacrifice of time and the amount of personal trouble and inconvenience the proper undertaking of it would entail is great enough to scare off all but the most enthusiastic of researchers. Take cancer of the breast, for instance. The largest collection of analysed cases is undoubtedly that of Finsterer, already alluded to, and yet, as everyone acquainted with statistics will readily admit, it is absurd to draw more than vague general conclusions from a summary of only some 700 cases. An investigator capable of undertaking the work in London and the large provincial centres will find records of several thousand cases available for his lists, and the collected results will be of infinitely greater comparative importance. The investigation will take time, and it will entail trouble. For it will mean the careful and laborious tracing of every case to the grave, or to a personal, or at least properly controlled, examination. Cases in which the patients died should be as carefully investigated as cases in which they are still alive, for it is of the utmost importance to know as fully as possible the percentage of recurrences and the actual mortality after operation. Such an investigation should prove fruitful in surprises. The statistical method is doubtless dry and dusty, but it is a very valuable method when properly used, and there are times when its results can kill a theory with mathematical exactitude. And it is well that theories should be weighed by clinical statistics. In some cases that criterion is the only one that can be usefully applied. But in all cases the statistical method should be patiently and carefully used, and by investigators who have a knowledge of their tools. It is not a matter of mere technique, but of discrimination and logical reasoning. Every qualified man may be able to do cancer research in the laboratory, but few, we feel convinced, will be able to do it properly in the registrar's room. Nevertheless it is a method of helping towards the solution of the problem which should not be lost sight of, and we cordially hope that in the near future it may be enthusiastically taken up. In this matter Prof. Pearson and his colleagues may well come to the aid of the mere medical man. At any rate no one who undertakes cancer research in the registrar's room on a large scale should neglect to profit by the lessons which the pioneers of eugenics have already taught us.

